# Adaptive Evolution of the Spike Protein in Coronaviruses

**DOI:** 10.1093/molbev/msad089

**Published:** 2023-04-13

**Authors:** Xiaolu Tang, Zhaohui Qian, Xuemei Lu, Jian Lu

**Affiliations:** State Key Laboratory of Protein and Plant Gene Research, Center for Bioinformatics, School of Life Sciences, Peking University, Beijing, China; NHC Key Laboratory of Systems Biology of Pathogens, Institute of Pathogen Biology, Chinese Academy of Medical Sciences and Peking Union Medical College, Beijing, China; State Key Laboratory of Genetic Resources and Evolution/Yunnan Key Laboratory of Biodiversity Information, Kunming Institute of Zoology, Chinese Academy of Sciences, Kunming, China; University of Chinese Academy of Sciences, Beijing, China; State Key Laboratory of Protein and Plant Gene Research, Center for Bioinformatics, School of Life Sciences, Peking University, Beijing, China

**Keywords:** SARS-CoV-2, coronavirus, Spike protein, molecular evolution, positive selection, adaptive evolution

## Abstract

Coronaviruses are single-stranded, positive-sense RNA viruses that can infect many mammal and avian species. The Spike (S) protein of coronaviruses binds to a receptor on the host cell surface to promote viral entry. The interactions between the S proteins of coronaviruses and receptors of host cells are extraordinarily complex, with coronaviruses from different genera being able to recognize the same receptor and coronaviruses from the same genus able to bind distinct receptors. As the coronavirus disease 2019 pandemic has developed, many changes in the S protein have been under positive selection by altering the receptor-binding affinity, reducing antibody neutralization activities, or affecting T-cell responses. It is intriguing to determine whether the selection pressure on the *S* gene differs between severe acute respiratory syndrome coronavirus 2 (SARS-CoV-2) and other coronaviruses due to the host shift from nonhuman animals to humans. Here, we show that the *S* gene, particularly the S1 region, has experienced positive selection in both SARS-CoV-2 and other coronaviruses. Although the S1 N-terminal domain exhibits signals of positive selection in the pairwise comparisons in all four coronavirus genera, positive selection is primarily detected in the S1 C-terminal domain (the receptor-binding domain) in the ongoing evolution of SARS-CoV-2, possibly owing to the change in host settings and the widespread natural infection and SARS-CoV-2 vaccination in humans.

## Introduction

Coronaviruses are single-stranded, positive-sense RNA viruses that infect various mammal and avian species (Li 2016; Cui et al. 2019). Coronaviruses are divided into four genera, namely, *Alphacoronavirus* (α-CoV), *Betacoronavirus* (β-CoV), *Gammacoronavirus* (γ-CoV), and *Deltacoronavirus* (δ-CoV). Typically, α-CoVs and β-CoVs infect mammals, γ-CoVs infect birds, and δ-CoVs infect both mammals and birds (Li 2016). Prior to 2019, six human-infecting coronaviruses were known: human coronaviruses (HCoVs) 229E (Hamre and Procknow 1966), NL63 (van der Hoek et al. 2004), HKU1 (Woo et al. 2005), OC43 (McIntosh et al. 1967), severe acute respiratory syndrome coronavirus (SARS-CoV) (Drosten et al. 2003; Ksiazek et al. 2003; Peiris et al. 2003), and Middle East respiratory syndrome coronavirus (MERS-CoV) (Zaki et al. 2012). Although HCoV-229E, HCoV-NL63, HCoV-HKU1, and HCoV-OC43 cause the common cold, infection by SARS-CoV and MERS-CoV, both of which are β-CoVs, can result in pneumonia and even death. SARS-CoV-2, the etiological agent of coronavirus disease 2019 (COVID-19) (Ren et al. 2020; Wu et al. 2020; Zhou et al. 2020), also belongs to the β-CoVs.

**Fig. 1.**
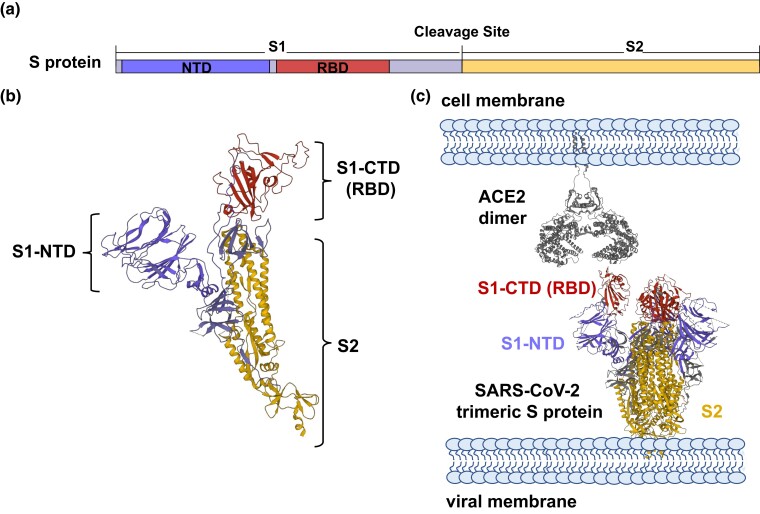
The schematic diagram of the S proteins of SARS-CoV-2. (*a*) Domain delineation based on SARS-CoV-2's S protein. NTD, N-terminal domain; RBD, receptor-binding domain. SARS-CoV-2's C-terminal domain (CTD) functions as RBD. (*b*) Monomer structure of SARS-CoV-2's S protein (PDB: 6ZGG) (Wrobel et al. 2020). (*c*) The binding between human ACE2 dimer (PDB: 6M17) and the trimeric S protein of SARS-CoV-2 (PDB: 6VYB) (Cai et al. 2020).

The Spike (S) protein of coronaviruses forms a trimer and initiates viral entry by interacting with a receptor on the host cell surface to enact virus attachment and induce membrane fusion (Du et al. 2009; Li 2016). The S protein is subdivided into S1 and S2, corresponding to residues 1–685 and 686–1,273, respectively, in the S protein of SARS-CoV-2 (fig. 1*a*). S1 recognizes and binds to the receptor expressed on the host cell surface, and S2 fuses the membranes of the virus and host cells (Li 2016). S1, which plays an essential role in determining host ranges and tissue tropism, is further divided into the N-terminal domain (S1-NTD) and the C-terminal domain (S1-CTD), either of which can function as the receptor-binding domain (RBD) to induce cellular entry (fig. 1*a*). When functioning as the RBD, S1-NTD typically binds sialic acid on the host cell surface to trigger viral entry into host cells (Li 2016). Occasionally, S1-NTD binds to a protein receptor of host cells for viral entry (Kubo et al. 1994). S1-CTD usually binds protein receptors, although the specific receptor may vary among coronaviruses. For instance, by using S1-CTD as the RBD, in the α-CoV genus, HCoV-NL63 binds ACE2, but transmissible gastroenteritis virus (TGEV), porcine epidemic diarrhea virus (PEDV), and porcine respiratory coronavirus (PRCV) recognize APN; in the β-CoV genus, SARS-CoV binds ACE2, while HCoV-HKU4 and MERS-CoV recognize DPP4 (Li 2016). Similar to SARS-CoV (Li et al. 2003), SARS-CoV-2 binds to the ACE2 receptor using S1-CTD of the S protein as the RBD to enter cells (Cai et al. 2020; Lan et al. 2020; Ou et al. 2020; Wang et al. 2020; Wrobel et al. 2020) (fig. 1*b* and *c*). S1-NTD of SARS-CoV-2 does not directly bind to ACE2, but it can bind to specific host protein coreceptors, such as LDLRAD3, TMEM30A, CLEC4G, and AXL, to facilitate viral entry (Wang et al. 2021; Zhu et al. 2022). Overall, the interactions between the S proteins of coronaviruses and receptors of host cells are extraordinarily complex, with coronaviruses from different genera being able to recognize the same receptor and coronaviruses from the same genus able to bind distinct receptors.

As the COVID-19 pandemic has developed, thousands of SARS-CoV-2 mutations have arisen (Qian et al. 2022). However, changes in the *S* gene have drawn the most attention since the sequence of the S protein influences the virus's ability to infect cells and evade host immune responses (Harvey et al. 2021). Population genetic studies have shown that the D614G amino acid change in the S protein of SARS-CoV-2 is driven by positive selection (Korber et al. 2020; Tang et al. 2021; Trucchi et al. 2021; Volz et al. 2021; Ruan et al. 2022). Experimental data also showed that the D614G mutation increases viral replication and transmissibility (Hou et al. 2020; Hu et al. 2020; Plante et al. 2020; Yurkovetskiy et al. 2020; Zhang et al. 2020; Daniloski et al. 2021; Ozono et al. 2021). In addition to D614G, many other changes in the S protein were under positive selection by altering the receptor-binding affinity, reducing antibody neutralization activities, or affecting T-cell responses (Harvey et al. 2021; Rochman et al. 2021; Qian et al. 2022). For instance, the currently recognized variants of interest (VOIs) and variants of concern (VOCs) SARS-CoV-2 lineages are primarily defined by mutations in the *S* gene (Harvey et al. 2021; Qian et al. 2022).

Since the majority of the coronaviruses use nonhuman animals as hosts, we hypothesize that the different host environments may have led to alterations in the target sites of positive selection in the *S* gene after SARS-CoV-2 recently invaded humans. This hypothesis is based on several considerations. First, the antibody-mediated immune responses may be weaker in nonhuman animals than in humans (Schountz et al. 2017). Second, the increase in the viral population size has played an important role in triggering the occurrences of adaptive mutations in SARS-CoV-2 (Ruan et al. 2022), and given that SARS-CoV-2 has infected an unprecedentedly large number of humans, this increase in population size may have contributed to the emergence of new variants (as of October 3, 2022, there have been 615,310,890 confirmed cases of COVID-19, https://covid19.who.int/). Third, the widespread use of vaccines (Dai et al. 2020; Dong et al. 2020; Dai and Gao 2021) may impose strong selective pressure on the full-length S protein or RBD of SARS-CoV-2 (Greaney et al. 2021a, 2021b), potentially accelerating the emergence and spread of new variants with mutations that confer immune escape.

Here, we show that the *S* gene, particularly the S1 region, has undergone substantial positive selection in both SARS-CoV-2 and other coronaviruses. Although S1-NTD exhibits positive selection in all four coronavirus genera, positive selection was primarily detected in S1-CTD (RBD) in the ongoing evolution of SARS-CoV-2, possibly owing to the change in host settings and the widespread natural infection and SARS-CoV-2 vaccination in humans.

## Results

### Positive Selection on the *S* Gene of SARS-CoV-2

**Fig. 2.**
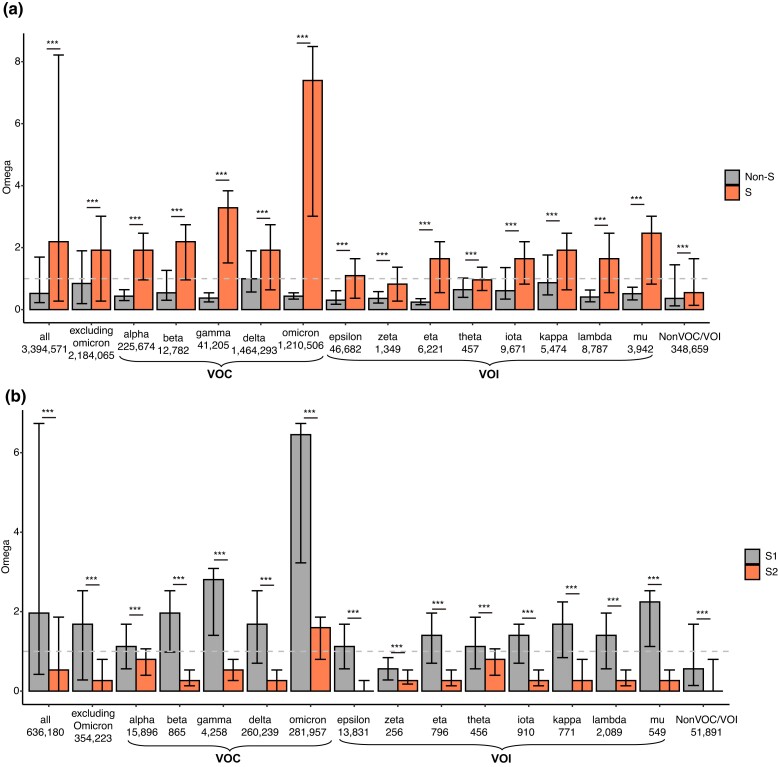
The *ω* values of *S* and non-*S* genes between SARS-CoV-2 strains and the reference genome. (*a*) The dN/dS (*ω*) value was significantly higher for the *S* gene than for the remaining genes when all the ORFs except *S* were concatenated. The 95% confidence intervals are presented. The bottom labels indicate the number of sequences with at least one synonymous change inside and outside the *S* gene. (*b*) The *ω* value was significantly higher in the S1 region than in the S2 region in the VOC/VOI lineage. The bottom labels indicate the number of sequences with at least one synonymous change inside both S1 and S2 regions. ***, *P* < 0.001.

To detect the signals of positive selection on the *S* gene in the continuing evolution of SARS-CoV-2, we downloaded 7,269,791 high-quality SARS-CoV-2 genomes from the GISAID database (http://gisaid.org, as of February 27, 2022). We calculated the dN (nonsynonymous substitutions per nonsynonymous site) and dS (synonymous substitutions per synonymous site) values between a particular genome and the reference genome (NC_045512) of SARS-CoV-2. We focused only on the SARS-CoV-2 genomes with at least one synonymous change inside and outside the *S* gene. With these criteria, we preserved 3,394,571 viral genomes in the analysis. As illustrated in fig. 2*a*, the dN/dS (*ω*) value was significantly higher for the *S* gene (the median, 2.5th, and 97.5th percentiles were 2.191, 0.274, and 8.215, respectively) than that for the concatenated sequences of the remaining genes (the median, 2.5th, and 97.5th percentiles were 0.523, 0.226, and 1.694, respectively) (*P* < 10^−10^, Wilcoxon signed rank test [WSRT]). Of note, the *ω* of *S* gene was significantly higher than 1 (*P* < 10^−10^, one-tailed one-sample WSRT), indicating positive selection; whereas the *ω* of non-*S* gene was significantly <1 (*P* < 10^−10^, one-tailed one-sample WSRT), indicating purifying selection. These findings support the notion that positive selection has driven protein sequence evolution in the *S* gene and that purifying selection is the dominant evolutionary force on the protein sequences of the remaining genes of SARS-CoV-2.

As the COVID-19 pandemic continued, the World Health Organization (WHO) identified VOCs and VOIs of SARS-CoV-2 with specific genetic markers potentially linked to increased viral transmissibility, disease severity, and immune evasion capabilities. To examine whether the *S* gene of different variant lineages demonstrates distinct positive selection strengths, we calculated the *ω* values between the reference genome (NC_045512) and each VOC/VOI lineage. As of October 3, 2022, there is only one circulating VOC (Omicron), four previously circulating VOCs (Alpha, Beta, Gamma, and Delta), and eight previously circulating VOIs (Lambda, Mu, Epsilon, Zeta, Eta, Theta, Iota, and Kappa). The phylogenetic analysis suggested that these VOC/VOI lineages tend to be independent of each other (supplementary fig. S1, Supplementary Material online). Supporting the notion that the VOCs/VOIs might have higher viral transmissibility or immune escape capabilities, the *ω* value was significantly higher in the *S* than in non-*S* genes between NC_045512 and all the VOC and VOI lineages (*P* < 0.001 in each comparison, WSRT; fig. 2*a*). Notably, the *ω* values were significantly >1 for the *S* gene between the reference genome and all five VOC variants (*P* < 10^−10^ in each comparison, one-tailed one-sample WSRT), with the median *ω* values in the order of Alpha (1.917) = Delta (1.917) < Beta (2.191) < Gamma (3.286) < Omicron (7.394) (fig. 2*a*). The higher *ω* value for the *S* gene of the Omicron lineage is consistent with the lineage's higher prevalence. The *ω* values of the *S* gene were significantly higher in the pooled VOC strains compared with the pooled VOI strains (*P* < 10^−10^, Wilcoxon rank-sum test). However, some VOI lineages, such as Mu (the median *ω* value was 2.465), Kappa (1.917), Lambda (1.643), Iota (1.643), and Eta (1.643), exhibited a *ω* value for the *S* gene that was significantly higher than 1 (*P* < 10^−10^ in each comparison, one-tailed one-sample WSRT). Additionally, the *ω* value was marginally but significantly higher in the *S* than that in non-*S* genes in the remaining SARS-CoV-2 strains (excluding VOCs and VOIs). Although these results suggest that VOC strains of SARS-CoV-2 underwent stronger positive selection in the *S* gene compared with VOI or other strains, which may have contributed to their higher prevalence in circulation, it is important to note that many other confounding factors can also affect the relative prevalence of a variant strain. These factors include vaccination and epidemic prevention policies in different countries and regions, as well as competition with other cocirculating strains.

To test whether selective strength varied across different regions of the *S* gene, we calculated the *ω* values of S1 and S2 for each SARS-CoV-2 genome (with at least one synonymous change inside both S1 and S2 regions) relative to the reference genome. Remarkably, the *ω* value was significantly higher in the S1 than in the S2 region in all SARS-CoV-2 genomes or VOC/VOI lineages (fig. 2*b*). Of note, the *ω* value of S1 was significantly >1 in each VOC lineage (*P* < 10^−10^ in each comparison, one-tailed one-sample WSRT; the median *ω* value was 1.122, 1.964, 2.806, 1.684, and 6.454 for the Alpha, Beta, Gamma, Delta, and Omicron lineages, respectively). In contrast, the *ω* of S2 was significantly <1 in the VOC lineages except for Omicron (*P* < 10^−10^ in each comparison, one-tailed one-sample WSRT; the median *ω* value was 0.798, 0.266, 0.532, 0.266, and 1.596 for the Alpha, Beta, Gamma, Delta, and Omicron lineages, respectively). These results suggest that the signal of positive selection in the *S* gene was primarily contributed by the nonsynonymous substitutions in the S1 region, while the amino acid changes in the S2 regions were overall under purifying selection except for in the Omicron lineage.

**Fig. 3.**
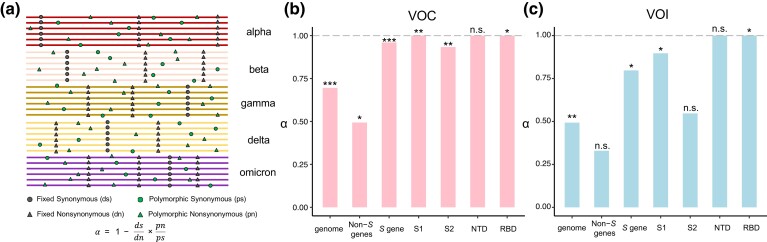
Detecting positive selection in the VOC and VOI lineages of SARS-CoV-2 using the McDonald–Kreitman test. (*a*) A scheme showing how the McDonald–Kreitman test was performed. Specifically, for each VOC/VOI, a mutation site with a frequency >0.8 was considered a fixed mutation, and a mutation site with a frequency between 0.01 and 0.8 was considered a polymorphic site. (*b*, *c*) According to the formula $\alpha \;=\;1\;-\;\frac{ds}{dn}\;\times \frac{\;pn}{\;ps}$, the *α* value of the genome, *S* gene, non-*S* gene, and individual region of the *S* gene was calculated in the VOC (*b*) and VOI (*c*) lineages. *, *P* < 0.05; **, *P* < 0.01; ***, *P* < 0.001; n.s., *P* > 0.05.

The above dN/dS (*ω*) analysis reveals the difference in selective pressure among S1, S2, and non-*S* genes of SARS-CoV-2. To confirm the signature of positive selection on S1 and to detect possible positive selection in other genomic regions, we carried out the McDonald–Kreitman test (Mcdonald and Kreitman 1991) on the VOC/VOI lineages. The McDonald–Kreitman test is more powerful in detecting positive selection even when protein sequences are under purifying selection (i.e., *ω* < 1). Briefly, we filtered mutations fixed in all VOCs and VOIs and considered only the polymorphic single-nucleotide polymorphisms (SNPs) across the VOC and VOI lineages. We counted the numbers of fixed synonymous sites (*ds*), polymorphic synonymous sites (*ps*), fixed nonsynonymous sites (*dn*), and polymorphic nonsynonymous sites (*pn*) in all the VOC or VOI lineages. Since clonal interference allows multiple beneficial mutations to coexist and compete with one another within a VOC/VOI lineage, we considered mutations with a frequency >0.8 within a variant lineage to be fixed. Since mutations with a frequency lower than 0.01 in each lineage are typically deleterious, we only considered polymorphic mutations with frequencies between 0.01 and 0.8 as neutral controls in the McDonald–Kreitman test. We calculated *α*, which is the proportion of fixed nonsynonymous mutations that are driven by positive selection in the divergence of the VOC or VOI lineages (fig. 3*a*). We detected positive selection signals in both the VOC linages (*α* = 0.696, *P* = 2.76 × 10^−6^, Fisher's exact test; fig. 3*b*) and VOI lineages (*α* = 0.493, *P* = 0.006; fig. 3*c*) when all the genes were pooled. Consistent with the notion that VOCs are more prevalent than VOIs, positive selection signals were stronger in the former than those in the latter (fig. 3*b* and *c*). Notably, the *S* gene showed strong signals of positive selection in both VOCs and VOIs (*α* = 0.96, *P* = 2.75 × 10^−5^ for VOCs, and *α* = 0.797, *P* = 0.009 for VOIs; fig. 3*b* and *c*). A significant positive selection signal was detected after excluding the *S* gene in the genomes of VOCs (*P* = 0.02) but not in VOIs (*P* = 0.18). Within the *S* gene, signals of positive selection were detected in S1 in both VOCs (*P* = 0.001) and VOIs (*P* = 0.017), while the signal of positive selection in S2 was statistically significant for VOCs (*P* = 0.008) but not for VOIs (*P* = 0.438). Within S1, signals of positive selection were identified in S1-CTD (RBD) in both VOCs (*P* = 0.011) and VOIs (*P* = 0.042), but not in S1-NTD in either VOCs or VOIs (*P* > 0.05 in both cases). Similar patterns were observed when defining mutations with a derived frequency >0.9 within a variant lineage as fixed and those between 0.01 and 0.9 as neutral controls in the McDonald–Kreitman test (supplementary fig. S2, Supplementary Material online). Thus, these findings further confirm that the *S* gene of SARS-CoV-2, especially S1-CTD (RBD), has been positively selected during the COVID-19 pandemic.

Collectively, both the dN/dS and McDonald–Kreitman tests have provided strong evidence of positive selection in the *S* gene, particularly in S1-CTD (RBD) of SARS-CoV-2. The RBD of SARS-CoV-2 mediates viral entrance by interacting with the ACE2 receptor, and it is also a target of host organisms’ neutralizing antibodies (Greaney et al. 2021a, 2021b). It is likely that the positive selection events in the RBD of SARS-CoV-2 are pertinent to rapid viral transmission and enhanced escape from hosts’ immune responses.

### Faster Evolution of the *S* Gene in the Four Genera of Coronaviruses

**Fig. 4.**
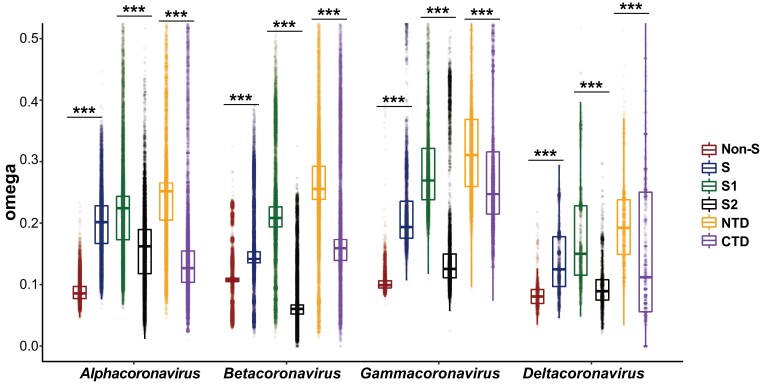
The dN/dS (*ω*) value of *S* and non-*S* genes in the pairwise comparisons in the four genera of coronaviruses. Only five genes (*orf1ab*, *S*, *E*, *M*, and *N*) that are present in all four coronavirus genera were used in the analysis. The values of the non-*S* genes were obtained by concatenating the CDS alignments of the four genes (*orf1ab*, *E*, *M*, and *N*). The S1-NTD and S1-CTD regions of the coronaviruses were annotated based on sequence alignments with the reference sequence of SARS-CoV-2 (S1-NTD corresponding to residues 13–304 and S1-CTD corresponding to residues 319–541 of the S protein of NC_045512). ***, *P* < 0.001.

To test whether the *S* gene has undergone adaptive evolution in the long-term evolution of coronaviruses, we downloaded the coronavirus genomes collected in the NCBI Virus Portal (in total, 490,630 genomes, last accessed November 17, 2021). We retained the genomes of 1,050 α-CoVs, 851 β-CoVs, 193 γ-CoVs, and 160 δ-CoVs in the analysis, after quality control and redundancy removal. Since the gene compositions varied considerably in the coronavirus genomes (Cui et al. 2019), we focused on the five genes present in all four coronavirus genera, namely, *orf1ab*, *S*, *E*, *M*, and *N*. In each genus, we calculated the pairwise dS, dN, and *ω* values of the *S* gene and the non-*S* genes (by concatenating coding alignments of the *orf1ab*, *E*, *M*, and *N* genes). In each genus, we considered only pairs of coronaviruses with the genomic dS values between 0.05 and 1 and dS values >0.05 in all the subregions of the *S* gene (S1, S2, NTD, and CTD) as well as in the non-*S* genes. This choice was made to achieve a balance between having sufficient synonymous mutations for accurate dS estimation and avoiding the generation of excessively large *ω* values caused by very small dS values. Finally, we preserved 44,104 pairs in α-CoVs, 56,229 pairs in β-CoVs, 14,290 pairs in γ-CoVs, and 2,668 pairs in δ-CoVs. In each genus, the ω values were significantly lower than 1 (P < 0.001, one-tailed one-sample WSRT; fig. 4), indicating that purifying selection is the dominant evolutionary force driving coronavirus evolution. Notably, *S* exhibited significantly higher *ω* values than non-*S* genes, and S1 showed significantly higher *ω* values than S2 in all the four genera (fig. 4). Furthermore, similar results were obtained when we used a dS cutoff value of 0.01 in estimating the *ω* values (supplementary fig. S3, Supplementary Material online). The most straightforward explanation for the observed higher *ω* values in the *S* gene, particularly in the S1 region, is that positive selection was the driving force behind coronavirus evolution in this gene. However, it should be noted that a relaxation of purifying selection could also contribute to these findings, although this alternative explanation is less probable (see below for further discussion).

Recombination has been frequently detected during the evolution of coronaviruses (Ji et al. 2020; Sun et al. 2020; Wu et al. 2020; Wertheim et al. 2022). To test whether the higher *ω* values in the *S* gene are due to recombination, we concatenated the aligned sequences of the five genes in each genus and performed recombination analysis using RDP4 (Martin et al. 2015). We employed seven algorithms (RDP, GENECONV, BootScan, MaxChi, Chimera, SiScan, and 3Seq) and identified 1,467 putative recombination events with at least one method (supplementary table S1, Supplementary Material online). To minimize the potential for false positives, we considered only those events identified by at least four of the seven algorithms as true recombination events. After excluding pairs of coronaviruses that had recombination events identified in the *S* gene by at least four algorithms, we still observed significantly higher *ω* values in the *S* than the non-*S* genes and significantly higher *ω* values for the S1 than the S2 region in all four genera of coronaviruses (supplementary fig. S4, Supplementary Material online). Therefore, the findings are not affected by recombination, supporting our conclusions.

### Positive Selection Targets the S1-NTD Region in Diverse Coronaviruses

The dN/dS and McDonald–Kreitman tests revealed substantial evidence of positive selection in S1-CTD (RBD) in both VOCs and VOIs of SARS-CoV-2 (fig. 3). In contrast, S1-NTD showed significantly higher ω values than S1-CTD in all four genera of coronaviruses (fig. 4), suggesting that S1-NTD was either under positive selection or relaxation of purifying selection during coronavirus evolution. To distinguish these two possibilities, we used CODEML (Yang 2007) to find positively selected sites in various coronavirus taxa and analyzed whether these sites were differentially positioned in the S1-NTD and S1-CTD regions.

To detect the signals of positive selection during the evolutionary divergence of SARS-CoV-2 and its closely related coronaviruses, we retrieved the genome sequences of 26 coronaviruses previously demonstrated to have high genetic similarity with SARS-CoV-2 (as presented in supplementary table S2, Supplementary Material online). We aligned the concatenated coding sequences (CDSs) of nine conserved ORFs for SARS-CoV-2 and the 26 coronaviruses and fitted the M7 and M8 models using CODEML as previously described (Tang et al. 2020). Comparing the M7 and M8 models, we detected a significant positive selection signal (*P* < 10^−10^) and identified 12 putative positively selected amino acid sites using Bayes empirical Bayes (BEB) analysis (supplementary table S3, Supplementary Material online). All 12 positively selected sites were located in the S1 region of the *S* gene, with 2 sites in the signal peptides, 9 sites in S1-NTD, and 1 site in S1-CTD. Remarkably, after correcting for the length difference, S1-NTD showed a significantly higher density of positively selected sites than S1-CTD (9/292 vs. 1/223, *P* = 0.049, Fisher's exact test). These findings indicate that the S1-NTD region of the *S* gene is the main target of positive selection in the evolutionary divergence of coronaviruses closely related to SARS-CoV-2.

To investigate whether similar patterns of positive selection can be observed in coronaviruses across the four genera, we randomly selected 50 viruses from each genus (see supplementary table S4, Supplementary Material online for the genome used in the analyses), aligned the concatenated CDSs of five conserved ORFs (*orf1ab*, *S*, *E*, *M*, and *N*), and used CODEML to detect significant signals of positive selection in each genus. We chose to use 50 genomes in each genus due to the computational intensity of CODEML analysis. Therefore, by using a smaller number (50) of genomes, we were able to perform the analyses more efficiently without compromising the statistical power of the study. We annotated the S1-NTD and S1-CTD regions of these coronaviruses based on sequence alignments with the reference sequence of SARS-CoV-2 (NC_045512). We identified 47, 66, 73, and 16 positive selection sites in these α-CoVs, β-CoVs, γ-CoVs, and δ-CoVs, respectively (see supplementary table S5, Supplementary Material online for a summary of the results). Among these sites, 21, 56, 37, and 11 were located in S1 in the four genera; in contrast, the equivalent values were 0, 0, 1, and 4 for S2. These findings support the notion that S1 is more likely the target of positive selection than S2 in all four coronavirus genera. We also identified more positively selected amino acid sites in S1-NTD than in S1-CTD (16 vs. 3 in α-CoVs, 33 vs. 21 in β-CoVs, 21 vs. 13 in γ-CoVs, and 10 vs. 0 in δ-CoVs), reinforcing the hypothesis that S1-NTD is more likely to be positively selected than S1-CTD in coronaviruses.

### Positive Selection on S1-NTD of Coronaviruses Is Not Solely Driven by Receptor Binding

The choice of whether to use S1-NTD or S1-CTD as the RBD varies among different coronaviruses (Li 2016; Cui et al. 2019). To investigate whether the difference in positive selection strength between S1-NTD and S1-CTD is related to receptor binding, we analyzed the evolutionary patterns of the *S* gene within 13 coronavirus species for which it is known whether S1-NTD or S1-CTD can bind the host receptor (table 1). Specifically, we analyzed four coronaviruses using S1-NTD for receptor binding (NTD binding): bovine coronavirus (BCoV), murine hepatitis virus (MHV), HCoV-OC43, and avian infectious bronchitis virus (IBV). In contrast, eight coronaviruses use S1-CTD to bind host receptors (CTD binding): canine coronavirus (CCoV), feline coronavirus (FCoV), HCoV-229E, HCoV-NL63, bat coronavirus HKU4 (HKU4), MERS-CoV, SARS-CoV, and porcine Deltacoronavirus (PDCoV). Moreover, PEDV uses both S1-NTD and S1-CTD to bind receptors. For each of these coronaviruses, we retrieved the CDSs of the *S* gene and calculated the pairwise *ω* value between different strains of that coronavirus (Materials and Methods and supplementary table S6, Supplementary Material online). Within each coronavirus except SARS-CoV, we exclusively considered pairs of strains with dS values >0.05 in all the subregions of the *S* gene (S1, S2, NTD, and CTD). For SARS-CoV, we required the pairwise dS values to be >0.005 in all the subregions of the *S* gene due to the high sequence similarity between strains.

**Table 1.** Comparison of the *ω* Values of Different Regions of the *S* Gene in 13 Coronaviruse**s**.

Species	Genus	Major Host	Receptor	# of Sequences	# of Pairs	S1 (mean ± SE)	S2 (mean ± SE)	*P* ^a^ (S1 vs. S2)	NTD (mean ± SE)	CTD (mean ± SE)	*P* ^a^ (NTD vs. CTD)	Collection Dates
NTD binds the host receptor												
BCoV	*β*	Bovine	Sugar (Schultze et al. 1991; Peng et al. 2012)	56	326	0.17 ± 0.01	0.08 ± 0.01	<10^−16^	0.27 ± 0.01	0.16 ± 0.01	<10^−16^	1983–2020
HCoV-OC43	*β*	Human	Sugar (Kunkel and Herrler 1993; Peng et al. 2012)	36	241	0.44 ± 0.01	0.16 ± 0.01	<10^−16^	0.60 ± 0.01	0.45 ± 0.01	<10^−16^	1991–2021
MHV	*β*	Murine	CEACAM1(Dveksler et al. 1991; Williams et al. 1991)	6	12	0.18 ± 0.01	0.12 ± 0.01	<10^−3^	0.23 ± 0.01	0.18 ± 0.01	0.007	1994–2014
IBV	*γ*	Avian	Sugar (Cavanagh and Davis 1986)	158	10,636	0.28 ± 0.01	0.13 ± 0.01	<10^−16^	0.32 ± 0.01	0.27 ± 0.01	<10^−16^	1941–2020
CTD binds the host receptor												
CCoV	*α*	Canine	APN (Tusell et al. 2007)	12	56	0.32 ± 0.01	0.05 ± 0.01	<10^−10^	0.38 ± 0.02	0.17 ± 0.01	<10^−9^	1971–2020
FCoV	*α*	Feline	APN (Tusell et al. 2007)	61	1,567	0.16 ± 0.01	0.07 ± 0.01	<10^−16^	0.19 ± 0.01	0.11 ± 0.01	<10^−16^	1993–2018
HCoV-229E	*α*	Human	APN (Tusell et al. 2007)	37	70	0.67 ± 0.01	0.08 ± 0.01	<10^−12^	0.68 ± 0.02	0.78 ± 0.01	<10^−3^	1993–2020
HCoV-NL63	*α*	Human	ACE2 (Hofmann et al. 2005; Hofmann et al. 2006)	48	219	0.18 ± 0.01	0.06 ± 0.01	<10^−16^	0.26 ± 0.01	0.10 ± 0.01	<10^−16^	1983–2018
HKU4	*β*	Bat	DPP4 (Wang et al. 2014; Yang et al. 2014)	6	10	0.43 ± 0.09	0.08 ± 0.02	0.002	0.34 ± 0.09	0.71 ± 0.09	0.002	2005–2014
MERS-CoV	*β*	Human and camel	DPP4 (Raj et al. 2013)	24	57	0.65 ± 0.02	0.48 ± 0.02	<10^−6^	0.50 ± 0.01	0.79 ± 0.03	<10^−9^	2013–2018
SARS-CoV	*β*	Human	ACE2 (Li et al. 2003)	54	76	1.01 ± 0.03	0.40 ± 0.02	<10^−13^	0.96 ± 0.03	0.90 ± 0.03	0.005	2003–2004
PDCoV	*δ*	Porcine	APN (Li et al. 2018)	106	1,674	0.12 ± 0.01	0.10 ± 0.01	<10^−16^	0.16 ± 0.01	0.06 ± 0.01	<10^−16^	2004–2020
Both NTD and CTD bind the host receptor												
PEDV	*α*	Porcine	Sugar (Liu et al. 2015); APN (Tusell et al. 2007)	643	45,087	0.21 ± 0.01	0.16 ± 0.01	<10^−16^	0.23 ± 0.01	0.13 ± 0.01	<10^−16^	1977–2019

a Wilcoxon signed rank test (WSRT).

Within all 13 studied coronaviruses, the pairwise *ω* values were significantly lower than 1 for both S1 and S2 regions. However, S1 exhibited significantly higher *ω* values than S2 (table 1). Notably, we detected significantly higher *ω* values on S1-NTD than S1-CTD in the four coronaviruses that use S1-NTD to bind host receptors (BCoV, HCoV-OC43, MHV, and IBV). However, mixed results were obtained for the eight coronaviruses that use S1-CTD to bind host receptors: although S1-CTD showed significantly higher *ω* values than S1-NTD in three coronaviruses (HCoV-229E, HKU4, and MERS-CoV), an opposite pattern was observed in the other five coronaviruses (CCoV, FCoV, HCoV-NL63, SARS-CoV, and PDCoV). In addition, a higher *ω* value was found in S1-NTD than in S1-CTD for PEDV, which uses both S1-NTD and S1-CTD to bind host receptors. These findings support the notion that positive selection targets S1-NTD not only in coronaviruses that utilize it as the RBD but also in those that use S1-CTD as the RBD. Moreover, the binding between S1-CTD and the host receptor does not appear to impose a stronger positive selection on S1-CTD compared with S1-NTD in the coronaviruses. Therefore, it is likely that other factors besides receptor binding contribute to the positive selection on S1-NTD in these coronaviruses.

## Discussion

The COVID-19 pandemic provides a unique opportunity to study the evolution of SARS-CoV-2, particularly its S protein, as it spreads extensively among humans. Comparing SARS-CoV-2 evolution with that of other coronaviruses that primarily infect nonhuman animals is important for understanding the impact of host change and widespread vaccination on viral evolution. The S protein of coronaviruses plays an essential role in facilitating virus entry and determining viral host range and tissue tropism. The S1 domain in the S protein potentially recognizes a variety of protein receptors and various sugars on the host cell surface. The S protein can also serve as a critical epitope for neutralizing antibodies of host organisms. Thus, it is anticipated that the S protein will evolve rapidly due to the evolutionary arms race between viruses and host organisms. Our study provides evidence that the *S* gene has undergone strong positive selection during the evolution of SARS-CoV-2 and, on a larger scale, during the evolution of coronaviruses belonging to various genera. Although S1-NTD exhibited signals of positive selection in all four genera of coronaviruses, positive selection was primarily detected in the S1-CTD (RBD) domain during the ongoing evolution of SARS-CoV-2.

**Fig. 5.**
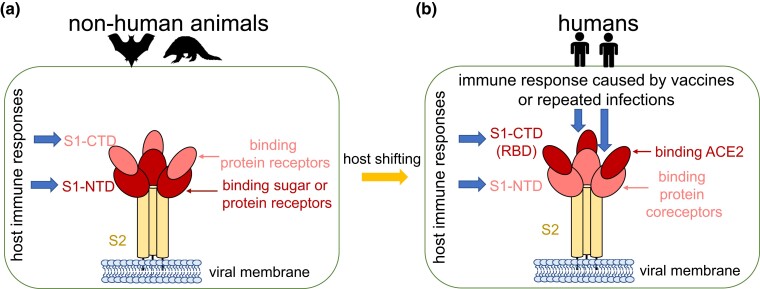
A model regarding how the selection pressure on the *S* gene differs between SARS-CoV-2 and other coronaviruses. (*a*) For coronaviruses that use nonhuman animal as hosts, either S1-NTD or S1-CTD can function as RBD to facilitate viral entry. When S1-NTD acts as RBD, it typically binds to sugar and occasionally to protein receptors on the host cells. When S1-CTD functions as RBD, it usually binds to protein receptors. The observed trend is that S1-NTD is more likely to be positively selected than S1-CTD, presumably driven by diversifying selection of receptor usage or by host immune responses. (*b*) S1-CTD (RBD) is more likely to be positively selected than S1-NTD after SARS-CoV-2 invades humans. The signal of positive selection in RBD of SARS-CoV-2 may be driven by the binding with human ACE2 receptor and the selective pressure of human immune responses elicited by repeated infections or the widespread SARS-CoV-2 vaccination in humans.

Coronaviruses use either S1-NTD or S1-CTD to bind host receptors. We detected significant signals of positive selection on S1-NTD throughout the evolution of coronaviruses, most of which use nonhuman animals as hosts (fig. 5*a*). S1-NTD primarily recognizes sugar receptors of host cells to facilitate virus entry. However, across coronaviruses, the interaction between S1-NTD and the sugar receptor frequently changes. For instance, in the α-CoV genus, TGEV and PEDV bind sugar receptors via S1-NTD. However, S1-NTD of TGEV recognizes both *N*-glycolylneuraminic acid (Neu5Gc) and *N*-acetylneuraminic acid (Neu5Ac), whereas S1-NTD of PEDV recognizes Neu5Ac, and S1-NTD of the γ-CoV IBV recognizes Neu5Gc (Li 2016). In addition to sugar receptors, S1-NTD can bind protein receptors (e.g., S1-NTD of the β-CoV MHV binds CEACAM1) or coreceptors (e.g., S1-NTD of SARS-CoV-2 potentially binds LDLRAD3, TMEM30A, CLEC4G, and AXL). Thus, the signal of positive selection detected in the S1-NTD throughout coronavirus evolution might result from diversifying selection of receptor usage, reflecting the coronaviruses’ continuous probing to adapt to different host species and tissues (Zehr et al. 2022). Moreover, S1-NTD, which can serve as an epitope for antibodies of host organisms that neutralize the coronaviruses (Chi et al. 2020; McCallum et al. 2021), may have evolved quickly due to the coronaviruses’ ability to evade host immune responses (fig. 5*a*).

Similar to S1-NTD, S1-CTD may have undergone rapid evolution due to 1) selective pressure to bind host receptors and 2) pressure to evade the host immune response. Interestingly, signals of positive selection were more likely to be detected in S1-NTD than S1-CTD when we performed pairwise comparisons between coronaviruses in each genus and when we compared SARS-CoV-2 with 26 closely related coronaviruses. In contrast, by comparing the genome sequences of various strains, we discovered that the signals of positive selection are mainly enriched in the S1-CTD (RBD) region of SARS-CoV-2 (fig. 5*b*). As we found inconsistent results when comparing the *ω* values of S1-NTD versus S1-CTD in the eight coronaviruses that use S1-CTD to bind host receptors, we reasoned that the positive selection on SARS-CoV-2 S1-CTD (RBD) is not necessarily associated with ACE2 binding.

Antibody-mediated immune responses in bats are not as strong as in humans (Schountz et al. 2017), which may have contributed to the observed positive selection in the S1-CTD of SARS-CoV-2 after its invasion into human populations, even though the origin of this virus remains unknown. However, our analysis of four other coronaviruses that affect humans showed mixed results (table 1), with some (HCoV-229E and MERS-CoV) exhibiting a higher *ω* value in S1-CTD than S1-NTD and others (HCoV-NL63 and SARS-CoV) exhibiting the opposite pattern, suggesting that host shift alone may not be sufficient to elicit positive selection on the S1-CTD of a coronavirus. In addition to the selective pressure for ACE2 binding and enhanced transmissibility, the selective pressures of the human immune system, including vaccine-induced immunity, have become increasingly important driving forces underlying SARS-CoV-2 evolution (Starr et al. 2020; Andreano et al. 2021; Cao et al. 2021, 2023; Greaney et al. 2021a, 2021b; Hu et al. 2021; Planas et al. 2021; Cao et al. 2022a, 2022b; Tan et al. 2022; Tegally et al. 2022).

Due to the fact that neutralizing antibodies elicited by natural infection or vaccination primarily target the S protein and the RBD region of SARS-CoV-2, the arms race between the virus and the immune system could lead to the emergence of viral variants with mutations in the *S* gene that confer immune escape. Given the unprecedented number of humans infected with SARS-CoV-2 and the billions of vaccine doses administered, many of which target the full-length S protein or RBD of SARS-CoV-2 (Dai et al. 2020; Dong et al. 2020; Dai and Gao 2021), adaptive mutations in S1-CTD (RBD) of SARS-CoV-2 have likely emerged to facilitate immune escape besides enhancing transmission (fig. 5*b*). The high mutation rate of SARS-CoV-2 (Chaw et al. 2020; Lai et al. 2020; Li et al. 2020; van Dorp et al. 2020), coupled with the strong selective pressure imposed by immune responses elicited by vaccines or natural infection, may accelerate the evolution of the *S* gene, particularly the RBD region of SARS-CoV-2. For instance, many independently evolved Omicron sublineages exhibit convergent mutations in the RBD region (Cao et al. 2023). As more individuals become infected or vaccinated, the selective pressures exerted by the immune responses on the virus may increase, further driving the evolution of SARS-CoV-2. Although vaccines play a crucial role in controlling the pandemic, mass vaccination may expedite the selection of new SARS-CoV-2 variants. However, additional research is necessary to determine the relative importance of vaccination versus natural infection in eliciting the immune pressure that drives the evolution of SARS-CoV-2. Continuous surveillance and research are required to ensure the efficacy of current vaccines and to guide future vaccine strategies.

## Materials and Methods

### Evolutionary Analysis of SARS-CoV-2 Genome Sequences

We downloaded 7,269,791 high-quality SARS-CoV-2 genomes from the GISAID database (http://gisaid.org, as of February 27, 2022). We aligned each genome sequence to the reference sequence (GenBank: NC_045512) using MAFFT v7.453 (−auto) (Katoh and Standley 2013). SnpEff v5.0e (Cingolani et al. 2012) was used to annotate the identified SNP. The numbers of synonymous (S) and nonsynonymous (N) sites for the concatenated non-*S* gene CDSs, *S* gene, S1, or S2 region of the reference genome were obtained by YN00 from PAML v4.9a (Yang 2007). For each SARS-CoV-2 genome, we calculated the dN, dS, and dN/dS (ω) values between that genome and the reference genome (NC_045512).

We used RAxMLv8.2.12 (Stamatakis 2014) to build a maximum likelihood phylogenetic tree of SARS-CoV-2 with the parameter “-p 1234 -m GTRCAT.” Interactive Tree Of Life (iTOL) v5 (https://itol.embl.de) was used to visualize the tree. To reduce the computational load in the phylogenetic reconstruction, we implemented a random subsampling strategy to achieve computational efficiency without compromising the statistical power of the phylogenetic reconstruction. Specifically, we randomly selected 1,000 genome sequences in a VOC/VOI lineage that had more than 1,000 sequences (Alpha, 1,046,682; Beta, 33,038; Gamma, 102,806; Delta, 3,695,538; Omicron, 1,297,803; Epsilon, 55,017; Zeta, 4,657; Eta, 6,742; Iota, 35,847; Kappa, 6,518; Lambda, 8,866; and Mu, 11,532) and included all available genome sequences in the lineage that had fewer than 1,000 genome sequences (Theta, 469, and VUM B.1.640, 533).

Using the McDonald–Kreitman test, we first filtered the mutations that were fixed for all VOCs and VOIs. For each VOC/VOI lineage, a mutation site with a frequency >0.8 is considered a fixed mutation, and a mutation site with a frequency between 0.01 and 0.8 is considered polymorphic. We counted the fixed synonymous sites (*ds*), polymorphic synonymous sites (*ps*), fixed nonsynonymous sites (*dn*), and polymorphic nonsynonymous sites (*pn*) in the VOCs and VOIs. Using the formula ($\alpha \;=\;1\;-\frac{ds}{dn}\;\times \frac{\;pn}{\;ps}$), we calculated the *α* value of the genome, *S* gene, non-*S* gene, and RBD region of SARS-CoV-2. We tested whether α is significantly different from 0 with Fisher's exact test for the four categories of mutations. Besides 0.8, we also defined mutations with a derived frequency >0.9 within a variant lineage as fixed and those between 0.01 and 0.9 as neutral controls in the McDonald–Kreitman test.

### The Pairwise Divergence between Coronaviruses in the Four Genera

The protein and genome sequences of the α-CoVs, β-CoVs, γ-CoVs, and δ-CoVs were downloaded from the NCBI Virus (https://www.ncbi.nlm.nih.gov/labs/virus/vssi/#/) database. Because the β-CoV group contains a large number of SARS-CoV-2 sequences, we used USEARCH (Edgar 2010) to remove redundant sequences (usearch11.0.667_i86linux64 -cluster_fast -id 0.996). Finally, 1,050 α-CoVs, 851 β-CoVs, 193 γ-CoVs, and 160 δ-CoVs were preserved in the analysis.

In each genus, MUSCLE v3.8.31 (Edgar 2004) was used to align the protein sequence of each of the five conserved genes (*orf1ab*, *S*, *E*, *M*, and *N*). RevTrans (Wernersson and Pedersen 2003) was used to align the CDSs based on the aligned protein sequence. YN00 from PAML v4.9a (Yang 2007) was used to calculate the pairwise dN, dS, and dN/dS (*ω*) values for each gene or the concatenated CDSs. The S1, S2, S1-NTD, and S1-CTD regions in the S protein of a coronavirus were parsed based on the homologous alignment to the S protein of SARS-CoV-2.

Recombination analysis was performed on the concatenated sequences of five genes (*orf1ab*, *E*, *M*, and *N*) using the RDP4 (Martin et al. 2015) package. Seven algorithms (RDP, GENECONV, BootScan, MaxChi, Chimera, SiScan, and 3Seq) were utilized to predict potential recombination events. We excluded from pairwise comparisons the parent–recombinant pairs of recombinant events detected by at least four programs in the *S* gene to eliminate the influence of recombination on pairwise divergence.

### Evolutionary Analysis of SARS-CoV-2 and 26 Closely Related Coronaviruses

We downloaded the sequences and annotations of the reference genome of SARS-CoV-2 and 26 coronaviruses that have been shown to be closely related to SARS-CoV-2 based on previous studies (supplementary table S2, Supplementary Material online) from GenBank (https://www.ncbi.nlm.nih.gov/genbank), GISAID (https://www.gisaid.org/), and Genome Warehouse (http://nmdc.cn/#/nCoV). The CDS sequences of the coronaviruses were parsed from the annotation information or identified based on the CDSs in SARS-CoV-2 annotated using Exonerate (−model protein2genome: bestfit –score 5 –g y) (Slater and Birney 2005). The protein sequences of SARS-CoV-2 and other related viruses were aligned with MUSCLE v3.8.31 (Edgar 2004), and codon alignments were made based on protein alignment with RevTrans (Wernersson and Pedersen 2003). The codon alignments of the nine ORFs homologous in SARS-CoV-2 and these coronaviruses (*orf1ab*, *S*, *ORF3a*, *E*, *M*, *ORF6*, *ORF7a*, *ORF7b*, and *N*) were concatenated for phylogenetic analysis. The phylogenetic tree was reconstructed by the neighbor-joining method (Saitou and Nei 1987) implemented in MEGA × software (Kumar et al. 2018) using the Jones‒Taylor‒Thornton (JTT) model (Jones et al. 1992). The pairwise deletion option was used to remove ambiguous sites.

### Detecting Positively Selected Amino Acid Sites Using CODEML

We used EasyCodeML (Gao et al. 2019), a wrapper of CODEML (Yang 2007), to detect the signal of positive selection. For each data set, the M7 (purifying selection and neutral evolution) and M8 (purifying selection, neutral evolution, and positive selection) models were contrasted. The likelihood ratio tests (LRTs) between the M7 and M8 models were performed by comparing twice the difference in log-likelihood values (2 ln ΔL) against a *χ*^2^ distribution (df = 2). The positively selected sites were identified by requiring the BEB score to be larger than 0.95.

### Pairwise Divergence between Strains in the 13 Coronaviruses

For pairwise comparisons of the *S* gene among distinct strains in the 13 coronaviruses (table 1), sequence divergence within each coronavirus was analyzed using procedures akin to the pairwise divergence analysis between strains within each genus. The detailed accession numbers for the sequences of strains within each coronavirus are provided in supplementary table S6, Supplementary Material online.

## Supplementary Material

msad089_Supplementary_Data

## Data Availability

The accession numbers of the sequences used in this study have been deposited into figshare (https://doi.org/10.6084/m9.figshare.22199386.v1). Any data and codes in this study are available from the corresponding author upon reasonable request.
