# Supplementary material for: Adaptive Evolution of the Spike Protein in Coronaviruses
Source: Mol Biol Evol. 2023 Apr 13;40(4):msad089. doi: 10.1093/molbev/msad089 (PMC10139704; doi:10.1093/molbev/msad089)
Supplement: msad089_Supplementary_Data [file msad089_supplementary_data.zip › Fast-evolution-S-Combined-MBE-SOM-Mar22_2023.pdf]

Supplementary tables and figures

Table S1. Recombination events detected in the four genera of coronaviruses

Genus	No. sequences	Recombination events (at least one algorithm)	Recombination events (at least four algorithms)	Recombination events (all seven algorithms)
Alphacoronavirus	1,050	376	214	65
Betacoronavirus	851	550	383	158
Gammacoronavirus	193	479	370	98
Deltacoronavirus	160	62	40	16

Table S2. SARS-CoV-2 and 26 closely related coronaviruses

Accession number	Database	Simplified name	Collection date	Location	Host	Ref
NC_045512	GenBank	SARS-CoV-2	2019-12	Asia/China/ Hubei	*Homo sapiens*	(Wu, et al. 2020)
MZ937000	GenBank	Bat BANAL-20-52	2020-07-05	Asia/Laos	*Rhinolophus malayanu*	(Temmam, et al. 2022)
MN996532	GenBank	Bat RaTG13	2013-07-24	Asia/China/ Yunnan	*Rhinolophus affinis*	(Zhou, Yang, et al. 2020)
MT040335	GenBank	Pangolin GX-P5 L	2017	Asia/China/ Guangxi customs	*Manis javanica*	(Lam, et al. 2020)
MZ937003	GenBank	Bat BANAL-20-236	2020-07-10	Asia/Laos	*Rhinolophus marshalli*	(Temmam, et al. 2022)
MZ937001	GenBank	Bat BANAL-20-103	2020-07-07	Asia/Laos	*Rhinolophus pusillus*	(Temmam, et al. 2022)
MT121216	GenBank	Pangolin GD MP789	2019-03-29	Asia/China/ Guangdong customs	*Manis javanica*	(Liu, et al. 2020)
EPI_ISL_852604	GISAID	Bat RSHSTT182	2010-12-06	Asia/Cambodia	*Rhinolophus shameli*	(Hul, et al. 2021)
EPI_ISL_852605	GISAID	Bat RSHSTT200	2010-12-06	Asia/Cambodia	*Rhinolophus shameli*	(Hul, et al. 2021)
MG772934	GenBank	Bat ZXC21	2015-07	Asia/China/ Zhejiang	*Rhinolophus pusillus*	(Hu, et al. 2018)
MG772933	GenBank	Bat ZC45	2017-02	Asia/China/ Zhejiang	*Rhinolophus pusillus*	(Hu, et al. 2018)
MW703458	GenBank	Bat PrC31	2018-08	Asia/China/ Yunnan	*Rhinolophus blythi*	(Li, et al. 2021)
LC556375	GenBank	Bat Rc-o319	2013	Asia/Japan	*Rhinolophus cornutus*	(Murakami, et al. 2020)
MZ937004	GenBank	Bat BANAL-20-247	2020-07-10	Asia/Laos	*Rhinolophus malayanu*	(Temmam, et al. 2022)
MZ937002	GenBank	Bat BANAL-20-116	2020-07-07	Asia/Laos	*Rhinolophus malayanu*	(Temmam, et al. 2022)
EPI_ISL_412977	GISAID	Bat RmYN02	2019-06-25	Asia/China/ Yunnan	*Rhinolophus malayanu*	(Zhou, Chen, et al. 2020)
MW251308	GenBank	Bat RacCs203	2020-06-19	Asia/Thailand	*Rhinolophus acuminatus*	(Wacharapluesadee, et al. 2021)
DQ412042	GenBank	Bat Rf1	2004	Asia/China/ Hubei	*Rhinolophus ferrumequinum*	(Li, et al. 2005)
KJ473812	GenBank	Bat Heb2013	2013-04	Asia/China/ Hebei	*Rhinolophus ferrumequinum*	(Wu, et al. 2016)
KF294457	GenBank	Bat Longquan-140	2012	Asia/China/ Zhejiang	*Rhinolophus monoceros*	(Lin, et al. 2017)
DQ022305	GenBank	Bat HKU3-1	2004-2005	Asia/China/ Hong Kong	*Rhinolophus sinicus*	(Lau, et al. 2005)
AY278488	GenBank	SARS BJ01	2003	Asia/China/ Beijing	*Homo sapiens*	(Wu, et al. 2003)
KF367457	GenBank	Bat WIV1	2012-09	Asia/China/ Yunnan	*Rhinolophus sinicus*	(Ge, et al. 2013)
KF569996	GenBank	Bat LYRA11	2011	Asia/China/ Yunnan	*Rhinolophus affinis*	(Hemida, et al. 2014)
KY352407	GenBank	Bat BtKY72	2007-08	Europe/Kenya	*Rhinolophus sp.*	(Tao and Tong 2019)
NC_014470	GenBank	Bat BM48-31	2008	Europe/Bulgaria	*Rhinolophus blasii*	(Drexler, et al. 2010)
GWHBAUP01000000	Genome Warehouse	Bat RaTG15	2015	Asia/China/ Yunnan	*Rhinolophus affinis*	(Guo, et al. 2021)

Reference:

Drexler JF, Gloza-Rausch F, Glende J, Corman VM, Muth D, Goettsche M, Seebens A, Niedrig M, Pfefferle S, Yordanov S, et al. 2010. Genomic characterization of severe acute respiratory syndrome-related coronavirus in European bats and classification of coronaviruses based on partial RNA-dependent RNA polymerase gene sequences. J Virol 84:11336-11349.

Ge XY, Li JL, Yang XL, Chmura AA, Zhu G, Epstein JH, Mazet JK, Hu B, Zhang W, Peng C, et al. 2013. Isolation and characterization of a bat SARS-like coronavirus that uses the ACE2 receptor. Nature 503:535-538.

Guo H, Hu B, Si HR, Zhu Y, Zhang W, Li B, Li A, Geng R, Lin HF, Yang XL, et al. 2021. Identification of a novel lineage bat SARS-related coronaviruses that use bat ACE2 receptor. Emerg Microbes Infect 10:1507-1514.

Hemida MG, Chu DK, Poon LL, Perera RA, Alhammadi MA, Ng HY, Siu LY, Guan Y, Alnaeem A, Peiris M. 2014. MERS coronavirus in dromedary camel herd, Saudi Arabia. Emerging Infectious Diseases 20:1231-1234.

Hu D, Zhu C, Ai L, He T, Wang Y, Ye F, Yang L, Ding C, Zhu X, Lv R, et al. 2018. Genomic characterization and infectivity of a novel SARS-like coronavirus in Chinese bats. Emerg Microbes Infect 7:154.

Hul V, Delaune D, Karlsson EA, Hassanin A, Ou Tey P, Baidaliuk A, Gambaro F, Tu VT, Keatts L, Mazet J, et al. 2021. A novel SARS-CoV-2 related coronavirus in bats from Cambodia. bioRxiv:2021.2001.2026.428212.

Lam TT, Jia N, Zhang YW, Shum MH, Jiang JF, Zhu HC, Tong YG, Shi YX, Ni XB, Liao YS, et al. 2020. Identifying SARS-CoV-2-related coronaviruses in Malayan pangolins. Nature 583:282-285.

Lau SK, Woo PC, Li KS, Huang Y, Tsoi HW, Wong BH, Wong SS, Leung SY, Chan KH, Yuen KY. 2005. Severe acute respiratory syndrome coronavirus-like virus in Chinese horseshoe bats. Proceedings of the National Academy of Sciences of the United States of America 102:14040-14045.

Li LL, Wang JL, Ma XH, Sun XM, Li JS, Yang XF, Shi WF, Duan ZJ. 2021. A novel SARS-CoV-2 related coronavirus with complex recombination isolated from bats in Yunnan province, China. Emerg Microbes Infect 10:1683-1690.

Li W, Shi Z, Yu M, Ren W, Smith C, Epstein JH, Wang H, Crameri G, Hu Z, Zhang H, et al. 2005. Bats are natural reservoirs of SARS-like coronaviruses. Science 310:676-679.

Lin XD, Wang W, Hao ZY, Wang ZX, Guo WP, Guan XQ, Wang MR, Wang HW, Zhou RH, Li MH, et al. 2017. Extensive diversity of coronaviruses in bats from China. Virology 507:1-10.

Liu P, Jiang JZ, Wan XF, Hua Y, Li L, Zhou J, Wang X, Hou F, Chen J, Zou J, et al. 2020. Are pangolins the intermediate host of the 2019 novel coronavirus (SARS-CoV-2)? Plos Pathogens 16:e1008421.

Murakami S, Kitamura T, Suzuki J, Sato R, Aoi T, Fujii M, Matsugo H, Kamiki H, Ishida H, Takenaka-Uema A, et al. 2020. Detection and characterization of bat sarbecovirus phylogenetically related to SARS-CoV-2, Japan. Emerg Infect Dis 26:3025-3029.

Tao Y, Tong S. 2019. Complete Genome Sequence of a Severe Acute Respiratory Syndrome-Related Coronavirus from Kenyan Bats. Microbiol Resour Announc 8.

Temmam S, Vongphayloth K, Salazar EB, Munier S, Bonomi M, Regnault B, Douangboubpha B, Karami Y, Chretien D, Sanamxay D, et al. 2022. Bat coronaviruses related to SARS-CoV-2 and infectious for human cells. Nature.

Wacharapluesadee S, Tan CW, Maneeorn P, Duengkae P, Zhu F, Joyjinda Y, Kaewpom T, Chia WN, Ampoot W, Lim BL, et al. 2021. Evidence for SARS-CoV-2 related coronaviruses circulating in bats and pangolins in Southeast Asia. Nat Commun 12:972.

Wu F, Zhao S, Yu B, Chen YM, Wang W, Song ZG, Hu Y, Tao ZW, Tian JH, Pei YY, et al. 2020. A new coronavirus associated with human respiratory disease in China. Nature 579:265-269.

Wu Q, Zhang Y, Lu H, Wang J, He X, Liu Y, Ye C, Lin W, Hu J, Ji J, et al. 2003. The E protein is a multifunctional membrane protein of SARS-CoV. Genomics Proteomics Bioinformatics 1:131-144.

Wu Z, Yang L, Ren X, He G, Zhang J, Yang J, Qian Z, Dong J, Sun L, Zhu Y, et al. 2016. Deciphering the bat virome catalog to better understand the ecological diversity of bat viruses and the bat origin of emerging infectious diseases. ISME J 10:609-620.

Zhou H, Chen X, Hu T, Li J, Song H, Liu Y, Wang P, Liu D, Yang J, Holmes EC, et al. 2020. A Novel Bat Coronavirus Closely Related to SARS-CoV-2 Contains Natural Insertions at the S1/S2 Cleavage Site of the Spike Protein. Curr Biol 30:3896.

Zhou P, Yang XL, Wang XG, Hu B, Zhang L, Zhang W, Si HR, Zhu Y, Li B, Huang CL, et al. 2020. A pneumonia outbreak associated with a new coronavirus of probable bat origin. Nature 579:270-273.

Table S3. The putative positively selected amino acid sites in the evolutionary divergence of SARS-CoV-2 and 26 closely related coronaviruses

Amino acid position on NC_045512	Gene	Domain	BEB score
L7	*S*	other regions of S1	1.000**
S12	*S*	other regions of S1	0.995**
Q23	*S*	NTD	0.982*
P25	*S*	NTD	0.977*
T33	*S*	NTD	0.991**
V70	*S*	NTD	0.999**
T76	*S*	NTD	0.993**
P85	*S*	NTD	0.995**
E154	*S*	NTD	0.996**
Q183	*S*	NTD	0.970*
Q218	*S*	NTD	0.988*
Q493	*S*	RBD	0.973*

The amino acid position is based on the coordinates relative to the protein in the SARS-CoV-2 reference genome (NC_045512). Although the amino acid in NC_045512 is presented for reference, it does not necessarily imply positive selection of the amino acid in SARS-CoV-2. * means the posterior probability is >95%, and ** means the probability is > 99%.

Table S4. The genome used in the CODEML analyses of the four genera of coronaviruses

Accession Number	Name
**α-CoVs**	
FJ938055	Feline coronavirus UU8
FJ938056	Feline coronavirus UU5
FJ938058	Feline coronavirus UU16
FJ938061	Feline coronavirus UU3
JQ989270	Rousettus bat coronavirus HKU10 isolate 183A
KC196276	Porcine epidemic diarrhea virus strain CH/ZMDZY/11
KF530108	Human coronavirus NL63 strain NL63/human/USA/891-6/1989
KF804028	Porcine epidemic diarrhea virus isolate USA/Iowa/18984/2013
KJ645638	Porcine epidemic diarrhea virus strain USA/Colorado30/2013
KJ645646	Porcine epidemic diarrhea virus strain USA/NorthCarolina40/2013
KJ645668	Porcine epidemic diarrhea virus strain USA/Minnesota73/2013
KJ645686	Porcine epidemic diarrhea virus strain USA/Minnesota94/2013
KM242131	Porcine epidemic diarrhea virus strain CH/GDZQ/2014
KM975738	Porcine epidemic diarrhea virus strain USA/IA/2013/19321
KP143510	Feline coronavirus isolate 67F
KR153325	Porcine epidemic diarrhea virus strain CH/GDZH02/1401
KR265759	Porcine epidemic diarrhea virus strain USA/Minnesota187/2014 from USA
KR265772	Porcine epidemic diarrhea virus strain USA/Kentucky248/2014 from USA
KR265820	Porcine epidemic diarrhea virus strain USA/Missouri337/2014 from USA
KR610991	Porcine epidemic diarrhea virus clone EAS1
KT253325	Camel alphacoronavirus Camel229E isolate Camel229E-CoV/JC49/KSA/2014
KU558701	Porcine epidemic diarrhea virus isolate ZJU/G2/2013
KU893866	Porcine epidemic diarrhea virus strain PC22A-P50
KU982979	Porcine epidemic diarrhea virus strain PEDV/USA/Minnesota131/2015
KX179500	Human coronavirus NL63 strain NL63/UF-2/2015
KX981440	Porcine epidemic diarrhea virus isolate CH/HNZZ47/2016
KY369913	Human coronavirus 229E strain HCoV_229E/Seattle/USA/SC1073/2016
KY499262	Porcine epidemic diarrhea virus strain PC22A
LC063822	Porcine epidemic diarrhea virus genomic RNA, strain: TTR-1/JPN/2014
LC063846	Porcine epidemic diarrhea virus genomic RNA, strain: MYZ-1/JPN/2013
MF373643	Porcine epidemic diarrhea virus
MH052682	Porcine epidemic diarrhea virus isolate KNU-1703
MH061341	Porcine epidemic diarrhea virus strain CH/SCLS/2018
MH687935	*Alphacoronavirus* sp. strain VZ_AlphaCoV_16715_24
MH687936	*Alphacoronavirus* sp. strain VZ_AlphaCoV_16715_31
MH687963	*Alphacoronavirus* sp. strain VZ_AlphaCoV_20724_95
MH726365	Porcine epidemic diarrhea virus isolate GDS25
MH726392	Porcine epidemic diarrhea virus isolate GDS44

MK071632	Porcine epidemic diarrhea virus strain COL/ValledelCauca/2015
MK392335	Porcine epidemic diarrhea virus isolate LW/L
MK409658	Porcine epidemic diarrhea virus isolate ZJ15XS0101-P35
MK644605	Porcine epidemic diarrhea virus isolate T10-HB2018
MK841494	Porcine epidemic diarrhea virus isolate PEDV SH
MT659926	Porcine epidemic diarrhea virus
MT683617	Porcine epidemic diarrhea virus strain MSCH
MT843280	Porcine epidemic diarrhea virus
MW165329	Porcine epidemic diarrhea virus isolate 4-2
MW202337	Human coronavirus NL63 isolate NL63_S170
MZ328299	Jingmen *Miniopterus schreibersii* alphacoronavirus 1
NC_032107	NL63-related bat coronavirus strain BtKYNL63-9a
**β-CoVs**	
AB551247	Murine hepatitis virus RNA, strain: MHV-MI
AY572038	SARS coronavirus civet020
AY772062	SARS coronavirus WH20
DQ415901	Human coronavirus HKU1 strain N24 genotype A
EF065506	Bat coronavirus HKU4-2
FJ588686	Bat SARS CoV Rs672/2006
KF530075	Human coronavirus OC43 strain OC43/human/USA/953-23/1995
KJ473814	BtRs-BetaCoV/HuB2013
KX442565	Hypsugo bat coronavirus HKU25 isolate NL140462
KY352407	Severe acute respiratory syndrome-related coronavirus strain BtKY72
KY417142	Bat SARS-like coronavirus isolate As6526
KY417149	Bat SARS-like coronavirus isolate Rs4255
KY419104	Porcine hemagglutinating encephalomyelitis virus strain PHEV-CoV/swine/USA/15TOSU0331/2015
KY967360	Human coronavirus OC43 strain HCoV_OC43/Seattle/USA/SC2476/2015
KY994645	Porcine hemagglutinating encephalomyelitis virus strain J.L./2008
LC315651	Human coronavirus HKU1 Tokyo/SGH-18/2016 RNA
LC494126	Bovine coronavirus GIF-1 RNA
LC494144	Bovine coronavirus IWT-20 RNA
LC494152	Bovine coronavirus IWT-26 RNA
LC494157	Bovine coronavirus SHG-4 RNA
LC494176	Bovine coronavirus TCG-17 RNA
LC556375	Severe acute respiratory syndrome-related coronavirus Rc-o319 RNA
LC654453	Human coronavirus OC43 Fukushima_H189_2018 RNA
MG772933	Bat SARS-like coronavirus isolate bat-SL-CoVZC45
MG923472	Middle East respiratory syndrome-related coronavirus isolate MERS-CoV camel/Nigeria/NS004/2015
MH043952	Bovine coronavirus isolate 4-17-03
MH432120	Middle East respiratory syndrome-related coronavirus isolate 2366
MH687974	*Betacoronavirus* sp. strain VZ_BetaCoV_20724_43
MN306041	Human coronavirus OC43 strain HCoV_OC43/Seattle/USA/SC0810/2019

MN982198	Bovine coronavirus isolate BCOV-China/SWUN/A1/2018
MN982199	Bovine coronavirus isolate BCOV-China/SWUN/A10/2018
MT782114	Mutant Bat SARS coronavirus HKU3 isolate icHKU3-SRBD
MW251308	Bat coronavirus RacCS203
MW703458	*Sarbecovirus* sp. isolate PrC31
MW719567	Sarbecovirus RhGB01
MW825987	SARS-CoV-2/human/USA/PA-CDC-STM-000033319/2021
MW891664	SARS-CoV-2/human/USA/TX-CDC-ASC210015770/2021
MZ024065	SARS-CoV-2/human/USA/OR-CDC-ASC210038591/2021
MZ081380	*Betacoronavirus* sp. RsYN04 strain bat/Yunnan/RsYN04/2020
MZ214262	SARS-CoV-2/human/USA/IN-CDC-ASC210069678/2021
MZ342366	SARS-CoV-2/human/USA/FL-CDC-LC0062194/2021
MZ571532	SARS-CoV-2/human/USA/IL-19196840/2021
NC_006213	Human coronavirus OC43 strain ATCC VR-759
OK017814	*Sarbecovirus* sp. isolate GD2017H
OK017834	*Sarbecovirus* sp. isolate HN2021E
OK017838	*Sarbecovirus* sp. isolate JX2021G
OK017844	*Sarbecovirus* sp. isolate JX2021O
OK017851	*Sarbecovirus* sp. isolate YN2016E
OK318940	Human coronavirus OC43 strain OC43/China/21/2019
**γ-CoVs**	
AY514485	Infectious bronchitis virus serotype California 99
EU022525	Turkey coronavirus isolate TCoV-540
EU111742	Coronavirus SW1
EU817497	Infectious bronchitis virus strain H52
FJ888351	Infectious bronchitis virus strain H120
FJ904714	Infectious bronchitis virus strain Cal 1995
FJ904718	Infectious bronchitis virus strain Conn46 1983
FJ904719	Infectious bronchitis virus strain Conn46 1991
FJ904721	Infectious bronchitis virus strain Mass41 1972
FJ904722	Infectious bronchitis virus strain Mass41 1979
FN430414	Infectious bronchitis virus ITA/90254/2005
GQ427175	Turkey coronavirus strain TCoV/IN-517/94
GQ504721	Infectious bronchitis virus strain Arkansas Vaccine
GQ504722	Infectious bronchitis virus strain Georgia 1998 pass8
JF732903	Infectious bronchitis virus strain Sczy3
KJ425486	Infectious bronchitis virus strain ck/CH/LDL/120557
KJ425488	Infectious bronchitis virus strain ck/CH/LHB/110825
KJ425489	Infectious bronchitis virus strain ck/CH/LHB/111172
KJ425498	Infectious bronchitis virus strain ck/CH/LHB/130642
KJ425499	Infectious bronchitis virus strain ck/CH/LHB/131118
KJ425506	Infectious bronchitis virus strain ck/CH/LHLJ/111050
KJ425508	Infectious bronchitis virus strain ck/CH/LHN/090909

KP036504	Infectious bronchitis virus strain ck/CH/LHB/130630
KP036505	Infectious bronchitis virus strain ck/CH/LJL/130925
KP118883	Infectious bronchitis virus strain ck/CH/LHB/121041
KP118885	Infectious bronchitis virus strain ck/CH/LSD/110857
KP118887	Infectious bronchitis virus strain ck/CH/LHB/140532
KP118892	Infectious bronchitis virus strain ck/CH/LLN/130101
KR608272	Infectious bronchitis virus isolate LDT3-A
KT736031	Infectious bronchitis virus isolate ck/CH/LDL/150434-I
KT736032	Infectious bronchitis virus isolate ckCHLDL150434-II
KY626045	Avian coronavirus strain Ma5
KY933089	Avian coronavirus strain 1148-A
MG913342	Avian coronavirus isolate AvCoV/Gallus gallus/Brazil/sample 38/2013 GI-11, complete genome
MH021175	Avian coronavirus strain D274
MK581204	Infectious bronchitis virus strain gammaCoV/Ck/Poland/255/1997
MK581205	Infectious bronchitis virus strain gammaCoV/Ck/Poland/548/2004
MK728875	Infectious bronchitis virus strain M41-CK
MK878536	Infectious bronchitis virus isolate GA9977/2019
MK957244	Infectious bronchitis virus strain PR05
MN128087	Infectious bronchitis virus strain TW2575/98vac
MN512438	Infectious bronchitis virus isolate IBV/Ck/Can/18-049707
MN548285	Infectious bronchitis virus strain CR88
MN548287	Infectious bronchitis virus strain H120
MN690608	Bottlenose dolphin coronavirus strain 37112-1
MT367412	Turkey coronavirus strain gammaCoV/Tk/Poland/G160/2016
MT701511	Infectious bronchitis virus isolate GD17/04
MT978193	Infectious bronchitis virus strain ck/CH/LDL/091022-K1
MW024789	Avian coronavirus IA1162/2020
MZ367369	Infectious bronchitis virus isolate IBV/chicken/Belgium/4134_001/2019
**δ-CoVs**	
JQ065042	Porcine coronavirus HKU15 strain HKU15-44
JQ065044	White-eye coronavirus HKU16 strain HKU16-6847
KJ462462	Porcine coronavirus HKU15 strain OH1987
KJ481931	Deltacoronavirus PDCoV/USA/Illinois121/2014 from USA
KJ567050	Porcine deltacoronavirus 8734/USA-IA/2014
KJ569769	Porcine coronavirus HKU15 strain IN2847
KJ584356	Porcine coronavirus HKU15 strain SD3424
KJ584358	Porcine coronavirus HKU15 strain PA3148
KJ601779	Deltacoronavirus PDCoV/USA/Illinois136/2014 from USA
KP757891	Porcine deltacoronavirus isolate CHN-HB-2014
KR265848	Porcine deltacoronavirus strain USA/Minnesota214/2014
KR265850	Porcine deltacoronavirus strain USA/Michigan448/2014
KR265853	Porcine deltacoronavirus strain USA/Minnesota/2013
KR265854	Porcine deltacoronavirus strain USA/Minnesota454/2014

KR265860	Porcine deltacoronavirus strain USA/Nebraska209/2014
KR265864	Porcine deltacoronavirus strain USA/Minnesota292/2014
KR265865	Porcine deltacoronavirus strain USA/Iowa459/2014
KU981062	Porcine deltacoronavirus strain NH isolate passage 10
KX022603	Porcine deltacoronavirus strain PDCoV/USA/Minnesota140/2015
KX022604	Porcine deltacoronavirus strain PDCoV/USA/Nebraska137/2015
KX361344	Porcine deltacoronavirus isolate P2_13_ST2_0313/PDCoV/0213/Thailand
KY293677	Porcine deltacoronavirus isolate CH/JXJGS01/2016
KY364365	Porcine deltacoronavirus isolate KNU16-07
KY513725	Porcine deltacoronavirus strain CH/Jiangsu/2014
KY926512	Porcine deltacoronavirus isolate KNU16-11
LC216915	Coronavirus HKU15 genomic RNA, strain: S582N
LC260041	Porcine deltacoronavirus genomic RNA, strain: IWT/JPN/2014
LC260042	Porcine deltacoronavirus genomic RNA, strain: MYZ/JPN/2014
LC260043	Porcine deltacoronavirus genomic RNA, strain: OKN/JPN/2014
LC260044	Porcine deltacoronavirus genomic RNA, strain: YMG/JPN/2014
MF431743	Porcine deltacoronavirus strain SD
MF642322	Porcine deltacoronavirus strain CHN/GS/2016/1
MF642324	Porcine deltacoronavirus strain CHN/GS/2017/1
MF948005	Porcine deltacoronavirus strain HB-BD, complete sequence
MG242062	Porcine deltacoronavirus isolate CHN-HeB1-2017
MG832584	Porcine deltacoronavirus isolate CHN-HN-1601
MG837130	Porcine deltacoronavirus isolate KNU16-07-P5
MK211169	Porcine deltacoronavirus strain CHN/Sichuan/2017
MN173779	Porcine deltacoronavirus isolate CHN-GX11-2018
MN781985	Porcine deltacoronavirus isolate CHzmd2019
MT663769	Porcine deltacoronavirus strain CHN-TS1-2019
MW196362	Mutant porcine deltacoronavirus strain USA/IL/2014/026PDV_P11
MW345815	Deltacoronavirus HNU1-2
MW345816	Deltacoronavirus HNU3
MW685622	Porcine deltacoronavirus isolate PDCoV/Haiti/Human/0081-4/2014
MW685623	Porcine deltacoronavirus isolate PDCoV/Haiti/Human/0256-1/2015
MW854634	Porcine deltacoronavirus isolate 104-553
MZ291567	Porcine deltacoronavirus strain OH-FD22 P7
NC_016995	Wigeon coronavirus HKU20
NC_039208	Porcine coronavirus HKU15 strain HKU15-155

Table S5. A summary of the positively selected amino acid sites in the four genera of coronaviruses

Amino acid position on NC_045512	Gene	Domain	BEB score
**α-CoVs**			
D953	orf1ab	nsp3	0.983*
Y955	orf1ab	nsp3	0.998**
Q956	orf1ab	nsp3	0.991**
E961	orf1ab	nsp3	0.984*
W979	orf1ab	nsp3	0.984*
D981	orf1ab	nsp3	0.993**
Q990~Q991	orf1ab	nsp3	0.989*
Q990~Q991	orf1ab	nsp3	0.982*
Q990~Q991	orf1ab	nsp3	0.999**
Q990~Q991	orf1ab	nsp3	0.994**
Q990~Q991	orf1ab	nsp3	0.986*
P1207	orf1ab	nsp3	0.964*
I1551	orf1ab	nsp3	0.958*
D1893	orf1ab	nsp3	0.969*
I1912	orf1ab	nsp3	0.978*
Q4397	orf1ab	nsp12	1.000**
F4399	orf1ab	nsp12	0.996**
V16	S	NTD	0.957*
Q23	S	NTD	0.992**
F59	S	NTD	0.991**
F59~S60	S	NTD	0.994**
N61	S	NTD	0.953*
S71	S	NTD	0.995**
G72	S	NTD	0.999**
T73	S	NTD	0.996**
N74	S	NTD	0.992**
K77	S	NTD	0.955*
P133~Q134	S	NTD	0.998**
P133~Q134	S	NTD	0.962*
N137	S	NTD	0.999**
S162	S	NTD	0.998**
N164	S	NTD	0.969*
L270~Q271	S	NTD	0.952*
T323	S	CTD	0.977*
E324~S325	S	CTD	0.978*
R328	S	CTD	0.962*
P631~T632	S	other regions of S1	0.968*
P631~T632	S	other regions of S1	0.973*
~1M	M	/	0.956*

T7~I8	M	/	0.958*
T7~I8	M	/	0.966*
T7~I8	M	/	0.960*
G215~D216	N	/	0.955*
Q241	N	/	0.986*
Q241~Q242	N	/	0.982*
L382~P383	N	/	0.966*
Q386	N	/	0.986*
**β-CoVs**			
D930~E931	orf1ab	nsp3	0.999**
D930~E931	orf1ab	nsp3	0.974*
D930~E931	orf1ab	nsp3	0.969*
L2044	orf1ab	nsp3	0.995**
P2046	orf1ab	nsp3	0.994**
V2047	orf1ab	nsp3	0.992**
C15~V16	S	NTD	0.974*
V16	S	NTD	0.951*
N17	S	NTD	1.000**
T20	S	NTD	0.997**
P25	S	NTD	0.999**
A27	S	NTD	1.000**
N30	S	NTD	0.998**
F32	S	NTD	0.969*
I68	S	NTD	0.975*
G72	S	NTD	0.987*
T73	S	NTD	0.995**
G75	S	NTD	1.000**
T76	S	NTD	0.995**
K77	S	NTD	0.985*
Y145	S	NTD	1.000**
H146	S	NTD	0.997**
K147	S	NTD	1.000**
N148	S	NTD	0.992**
N149	S	NTD	1.000**
S151~W152	S	NTD	0.956*
S151~W152	S	NTD	0.984*
E154	S	NTD	1.000**
S172	S	NTD	1.000**
Q173	S	NTD	1.000**
I197	S	NTD	0.999**
K206	S	NTD	0.966*
V213	S	NTD	0.997**
Q218	S	NTD	1.000**

L249	S	NTD	0.991**
S255	S	NTD	0.999**
S256	S	NTD	0.999**
G261	S	NTD	0.976*
A262	S	NTD	0.969*
R346	S	CTD	0.999**
D405	S	CTD	0.953*
S443	S	CTD	0.998**
K458	S	CTD	0.999**
S459	S	CTD	0.987*
C480~N481	S	CTD	0.999**
C480~N481	S	CTD	0.982*
C480~N481	S	CTD	0.999**
C480~N481	S	CTD	0.994**
C480~N481	S	CTD	0.980*
C480~N481	S	CTD	0.973*
C480~N481	S	CTD	0.981*
C480~N481	S	CTD	0.983*
C480~N481	S	CTD	0.977*
P490	S	CTD	0.994**
S494	S	CTD	0.984*
G496~F497	S	CTD	0.981*
Q498	S	CTD	0.999**
P499~T500	S	CTD	0.988*
G504~Y505	S	CTD	0.952*
G504~Y505	S	CTD	0.966*
Y660~E661	S	other regions of S1	0.956*
N679~S680	S	other regions of S1	0.962*
A2	M	/	0.995**
S4	M	/	0.974*
P13~R14	N	/	0.999**
P13~R14	N	/	1.000**
**γ-CoVs**			
A206	orf1ab	nsp2	0.954*
P380	orf1ab	nsp2	0.975*
S1102	orf1ab	nsp3	0.962*
Q1140	orf1ab	nsp3	0.991**
D1547	orf1ab	nsp3	0.992**
T1788	orf1ab	nsp3	0.998**
T1794	orf1ab	nsp3	0.996**
S1905	orf1ab	nsp3	0.977*
N2596	orf1ab	nsp3	0.990**
V2629	orf1ab	nsp3	0.996**

W2769	orf1ab	nsp4	0.998**
A2784	orf1ab	nsp4	1.000**
L2874	orf1ab	nsp4	0.999**
R3206	orf1ab	nsp4	1.000**
Q3332	orf1ab	nsp5	1.000**
G3478	orf1ab	nsp5	0.976*
R4402	orf1ab	nsp12	0.986*
V4403	orf1ab	nsp12	1.000**
V4406	orf1ab	nsp12	1.000**
L4411	orf1ab	nsp12	1.000**
K4439	orf1ab	nsp12	0.995**
A5877	orf1ab	nsp13	0.953*
R7085	orf1ab	nsp16	0.990*
N25	S	NTD	0.968*
L18	S	NTD	1.000**
S46	S	NTD	0.997**
V90	S	NTD	0.981*
T95	S	NTD	0.980*
K97	S	NTD	1.000**
G107	S	NTD	0.971*
T109	S	NTD	0.994**
N122	S	NTD	0.999**
T124	S	NTD	1.000**
V127	S	NTD	0.999**
K129	S	NTD	1.000**
V130	S	NTD	1.000**
A163	S	NTD	1.000**
N165	S	NTD	1.000**
N185	S	NTD	0.994**
K195~N196	S	NTD	0.986*
K202	S	NTD	1.000**
L244	S	NTD	1.000**
A260	S	NTD	0.952*
A260~G261	S	NTD	0.970*
S349	S	CTD	1.000**
V362	S	CTD	1.000**
Y365	S	CTD	1.000**
Y369	S	CTD	0.999**
K378	S	CTD	1.000**
S383	S	CTD	0.993**
D427	S	CTD	0.992**
Y489	S	CTD	1.000**
L492	S	CTD	1.000**

Q493	S	CTD	0.999**
N501	S	CTD	1.000**
Y505	S	CTD	1.000**
Q506	S	CTD	1.000**
Q628	S	other regions of S1	1.000**
N641	S	other regions of S1	0.989*
A653	S	other regions of S1	1.000**
S686	S	S2	1.000**
D72	E	/	1.000**
V75~	E	/	0.966*
A2	M	/	1.000**
D3	M	/	0.996**
D3~S4	M	/	0.975*
K15	M	/	1.000**
F66	N	/	0.985*
P80	N	/	1.000**
Y172~A173	N	/	0.996**
D340	N	/	0.958*
K372~K373	N	/	0.986*
D377	N	/	0.989*
**δ-CoVs**			
F32~T33	S	NTD	0.987*
F32~T33	S	NTD	0.969*
R34	S	NTD	0.953*
Y37	S	NTD	0.982*
I119	S	NTD	0.981*
S151	S	NTD	0.974*
T167	S	NTD	0.976*
Q183	S	NTD	0.994**
A260	S	NTD	0.955*
Y265	S	NTD	0.960*
T307	S	other regions of S1	0.976*
T693	S	S2	0.961*
S810	S	S2	0.970*
K1038	S	S2	0.971*
L1145	S	S2	0.957*
D81	N	/	0.971*

The amino acid position is based on the coordinates relative to the protein in the SARS-CoV-2 reference genome (NC_045512). Although the amino acid in NC_045512 is presented for reference, it does not necessarily imply positive selection of the amino acid in SARS-CoV-2. The symbol "~" means an insertion between two amino acids relative to the reference sequence of SARS-CoV-2; "/" means that no functional domain is specified. * means the posterior probability is >95%, and ** means the probability is > 99%.

Table S6 is an Excel file containing the accession numbers of the strains used in pairwise comparisons within 13 coronaviruses.

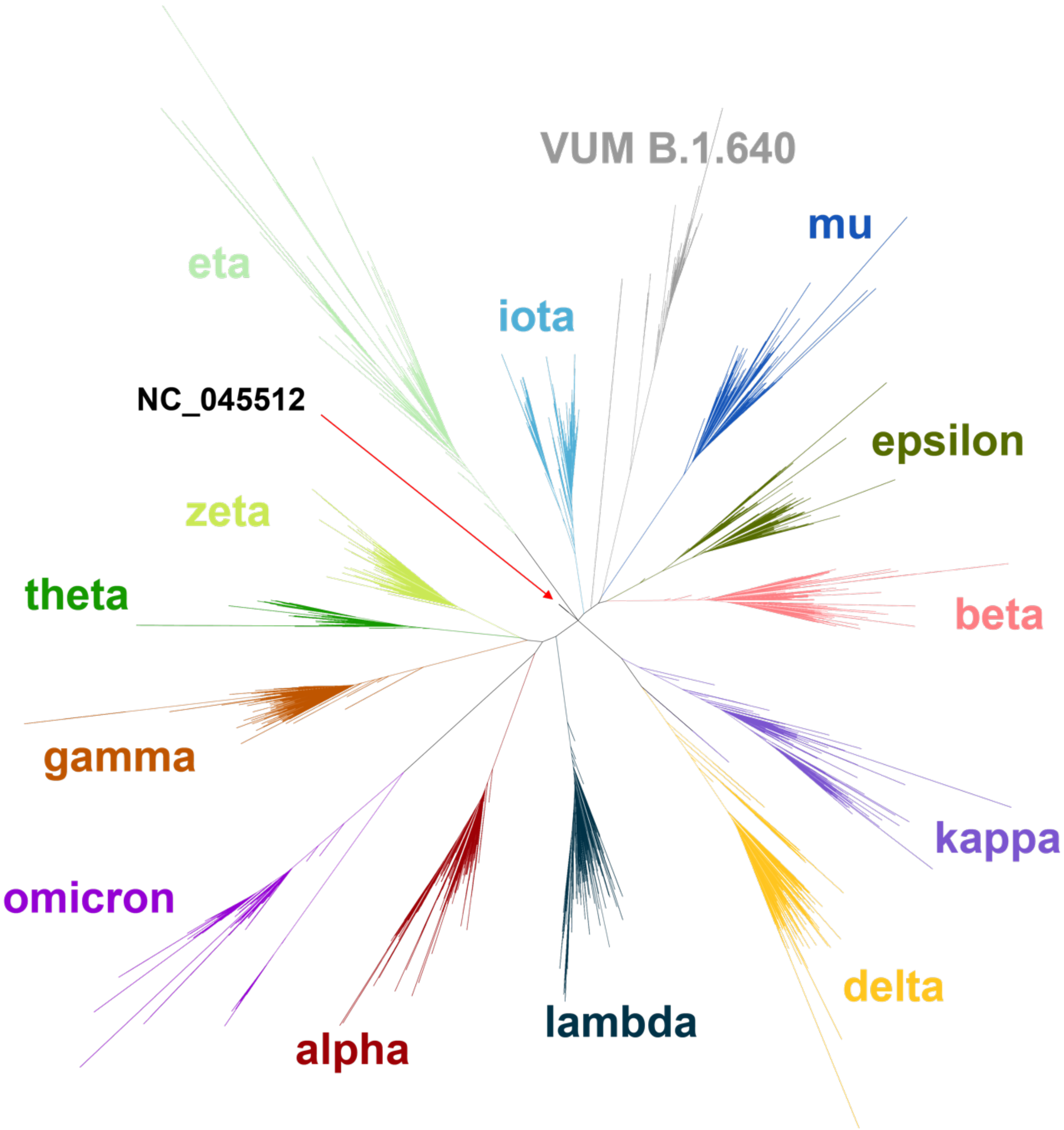

Fig. S1. The phylogenetic tree of SARS-CoV-2 VOCs and VOIs.

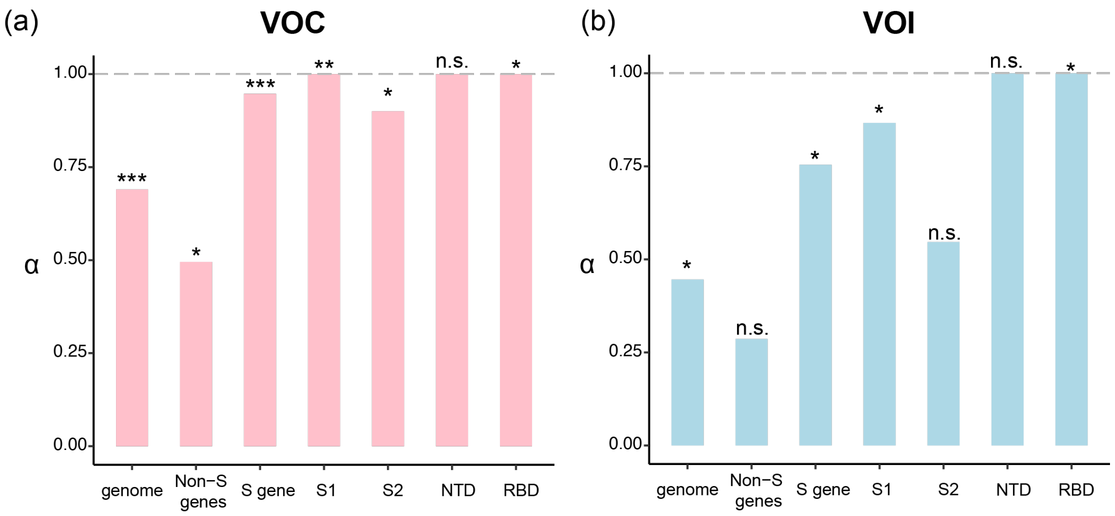

Fig. S2. The α value of the genome, *S* gene, non-*S* gene, and individual region of the *S* gene in the VOC (a) and VOI (b) lineages. For each VOC/VOI lineage, the mutations with a derived frequency greater than 0.9 within a variant lineage were defined to be fixed, while those between 0.01 and 0.9 were treated as neutral controls in the McDonald-Kreitman test. *, $P < 0.05$; **, $P < 0.01$; ***, $P < 0.001$; n.s., $P > 0.05$.

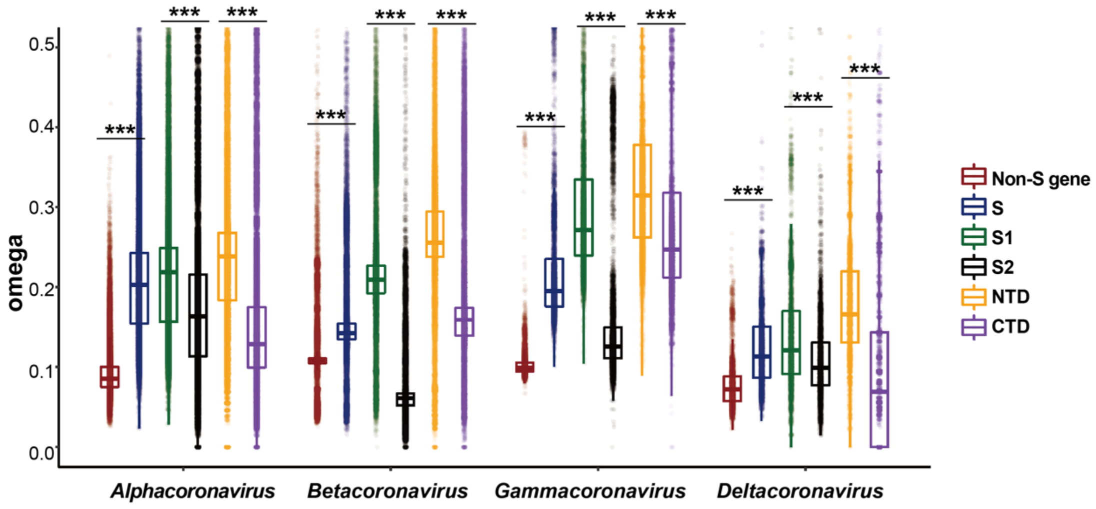

Fig. S3. The dN/dS (ω) value of *S* and non-*S* genes in the pairwise comparisons in the four genera of coronaviruses. In each genus, we only considered pairs of coronaviruses with dS values greater than 0.01 in all the sub-regions of the *S* gene (S1, S2, NTD, and CTD) as well as in the non-*S* genes. ***, $P < 0.001$.

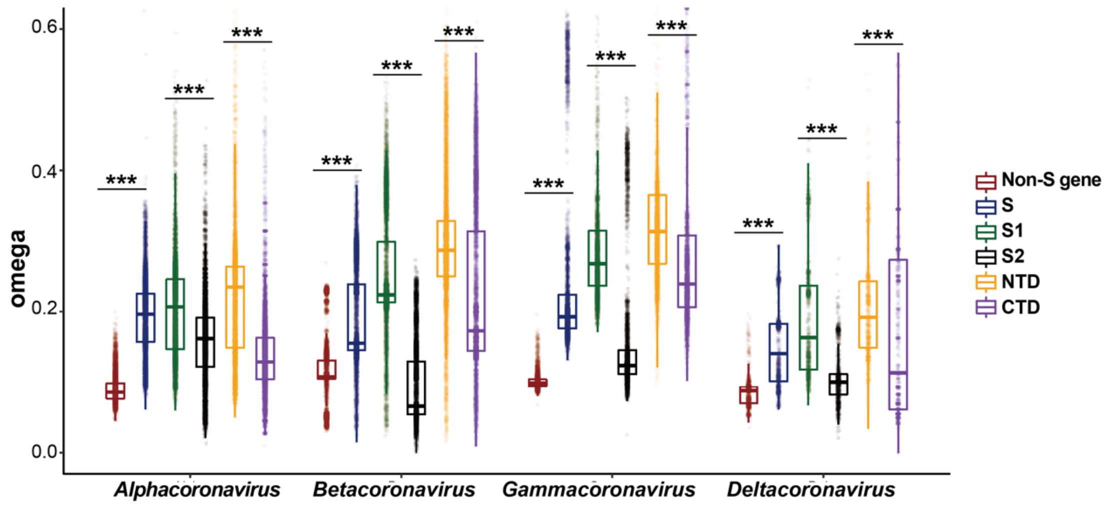

Fig. S4. The dN/dS (ω) value of *S* and non-*S* genes in the pairwise comparisons for the four genera after excluding the parent-recombinant pairs of recombination events that covered the *S* gene detected by at least four programs. ***, $P < 0.001$.
